# Parent and peer relationships as longitudinal predictors of adolescent non-suicidal self-injury onset

**DOI:** 10.1186/s13034-018-0261-0

**Published:** 2019-01-03

**Authors:** Sarah E. Victor, Alison E. Hipwell, Stephanie D. Stepp, Lori N. Scott

**Affiliations:** Department of Psychiatry, University of Pittsburgh, Sterling Plaza Suite 408, Pittsburgh, PA 15213 USA

**Keywords:** Non-suicidal self-injury, Parenting, Relationships, Family, Social, Adolescence, Discrete-time survival analysis, Longitudinal modeling

## Abstract

**Background:**

Adolescence is characterized by developmental changes in social relationships, which may contribute to, or protect against, psychopathology and risky behaviors. Non-suicidal self-injury (NSSI) is one type of risky behavior that typically begins during adolescence and is associated with problems in relationships with family members and peers. Prior research on social factors in adolescent NSSI has been limited, however, by a narrow focus on specific interpersonal domains, cross-sectional methods, retrospective self-report of childhood experiences, and a failure to predict NSSI onset among as-yet-unaffected youth.

**Methods:**

We investigated these relationships in 2127 urban-living adolescent girls with no NSSI history at age 13, who were participating in a longitudinal cohort study (Pittsburgh Girls Study). We used discrete-time survival analyses to examine the contribution of time-varying interpersonal risk factors, assessed yearly at ages 13–16, to NSSI onset assessed in the following year (ages 14–17), controlling for relevant covariates, such as depression and race. We considered both behavioral indicators (parental discipline, positive parenting, parental monitoring, peer victimization), and cognitive/affective indicators (quality of attachment to parent, perceptions of peers, and perceptions of one’s own social competence and worth in relation to peers) of interpersonal difficulties.

**Results:**

Parental harsh punishment, low parental monitoring, and poor quality of attachment to parent predicted increased odds of subsequent adolescent NSSI onset, whereas positive parenting behaviors reduced the odds of next year NSSI onset. Youth who reported more frequent peer victimization, poorer social self-worth and self-competence, and more negative perceptions of peers were also at increased risk of NSSI onset in the following year. When tested simultaneously, no single parenting variable showed a unique association with later NSSI onset; in contrast, peer victimization and poor social self-worth each predicted increased odds of later NSSI onset in an omnibus model of peer and parent relationship characteristics.

**Conclusions:**

In this urban sample of adolescent girls, both peer and parent factors predicted new onset NSSI, although only peer factors were associated with subsequent NSSI in combined multivariate models. Results further suggest that both behavioral and cognitive/affective indicators of interpersonal problems predict NSSI onset. These findings highlight the relevance of family and peer relationships to NSSI onset, with implications for prevention of NSSI onset among at-risk youth.

## Background

Non-suicidal self-injury (NSSI) is intentional, self-directed damage to body tissue without suicidal intent [1]. NSSI is common among adolescents, with lifetime prevalence rates of approximately 25% [2], and 1-year incidence rates of approximately 4% [3, 4]. In addition to the physical consequences of NSSI, these behaviors are associated with multiple types of psychopathology [5], particularly depression [6, 7] and increased risk of suicidal behavior [8, 9]. Importantly, even a single episode of NSSI is associated with impaired functioning and increased suicidality [10–12]. Thus, prevention of NSSI is an important public health concern. However, the majority of NSSI research has conflated predictors of onset of NSSI with correlates of increases or decreases in NSSI behaviors, due to the use of primarily cross-sectional data and/or longitudinal research with small samples. In addition, despite evidence that youth NSSI often occurs in response to interpersonal stressors [13] and can be reinforced by social factors [11, 14], there is a paucity of research examining both family and peer relationships as predictors of NSSI onset. To address these limitations, we focus on understanding parenting and peer-related risk factors for NSSI onset using prospectively collected data in a large urban sample of adolescent girls.

Research focused on identifying predictors of NSSI onset is necessary to elucidate key factors that identify at-risk individuals who might benefit from intervention to prevent, rather than treat, NSSI. This work is critical in light of evidence that correlates of new onset NSSI may be qualitatively different from correlates of continuing NSSI (or maintenance). For example, in a large, community-based sample of Australian youth, poorer perceived family support predicted both new onset NSSI and continued NSSI over a 1-year period; in contrast, low levels of support from a romantic partner or from friends predicted follow-up NSSI only for those already engaging in NSSI at baseline, but did not predict new onset NSSI [15]. Data from the same sample found that rumination also failed to show an association with subsequent NSSI onset [16], whereas prospective research among individuals already engaging in NSSI suggests that rumination contributes to continued engagement in NSSI [17]. Thus, existing research that fails to distinguish NSSI onset from maintenance may conflate the risk processes for these two phases of NSSI behavior.

Relationships with parents and peers, which are critical to adolescent mental health and well-being, represent one such area where we might expect to identify risk processes for NSSI onset. For example, poor quality of attachment to parents [18], harsh parental punishment [19], peer victimization [20], and low perceived social support [21] are strongly associated with depression and other internalizing problems, which are, in turn, associated with NSSI [22, 23]. Although family environment is likely to contribute to NSSI, for example, through expressed emotion [24], existing empirical and theoretical work on family factors as they relate prospectively to new onset of NSSI has been limited. There has also been extensive research on the possibility of NSSI “contagion” among adolescent peers [25]; evidence suggests, however, that few adolescents who know of friends’ NSSI actually report starting NSSI as a result of this knowledge [26]. Thus, more research is needed to clarify the interpersonal processes that contribute to NSSI onset in adolescence, in order to develop, test, and refine our theoretical models of NSSI.

Peer victimization is perhaps the most frequently investigated interpersonal risk factor for NSSI. Indeed, findings from a meta-analysis utilizing data from nine cross-sectional studies indicate that peer victimization is more common among youth who have engaged in NSSI compared to youth with no such history [27]. However, cross-sectional designs preclude inferences about the temporal ordering of these constructs. When evaluating longitudinal studies focused on peer victimization and NSSI, findings are mixed. In a systematic review, five studies reported a positive association between peer victimization and later NSSI, while two studies showed no evidence of this effect [28]. Interpretation of these findings is somewhat limited, however, as none specifically predicted new onset of NSSI, and the assessment of NSSI (presence/absence, frequency, number of methods) and follow-up timeframe varied across studies. Relatedly, negative views of school peers were associated with higher odds of lifetime engagement in NSSI [29], although this association has only been investigated using cross-sectional methods.

There has been some investigation of parent relationship factors in association with NSSI, although findings have been somewhat mixed, and longitudinal investigations have been sparse. For instance, in one study, quality of attachment to one’s parent was associated with history of NSSI [30], but this relationship was based on retrospective evaluation of adolescent attachment based on college student self-report. When assessed concurrently, parental monitoring has been unrelated to presence of NSSI [31], and also does not moderate the deleterious effects of peer victimization with respect to NSSI [32]. There is also cross-sectional evidence that family functioning may have indirect associations with NSSI through the connection between poor family functioning and depressive symptoms [33] and use of avoidance/emotion-focused coping [34], and that the relationship between NSSI and family functioning may be moderated by the extent to which parents are aware of their child’s NSSI [35]. Some longitudinal work suggests that harsh punishment predicts subsequent presence of NSSI [36], although this association has not been found in other samples [37]. This variability may be attributable to sex differences, as preliminary evidence suggests that harsh parenting predicts NSSI severity among adolescent girls but not boys [38]. There is conflicting research regarding the influence of positive parenting behaviors on NSSI, with some evidence suggesting positive parenting predicts *greater* subsequent odds of adolescent NSSI [39], and other research finding no such association [37]. Further, longitudinal research in the UK suggests that poor family functioning prospectively predicts new onset of NSSI during adolescence, and that family functioning mediates the association between childhood adversities and adolescent NSSI [40].

Existing research on interpersonal factors and NSSI has primarily focused on comparing individuals who are already engaging in NSSI to those without such a history; this work is likely to conflate potential interpersonal *contributors* to NSSI with interpersonal *correlates or consequences*. For example, research suggests that negative interpersonal life events prospectively predict NSSI [41]; however, there is also evidence indicating that engagement in NSSI predicts subsequent increases in these types of stressful events [42], consistent with models of stress generation in depression [43]. Even longitudinal research on NSSI has primarily focused on predicting changes in NSSI engagement (for example, frequency) over time among youth, rather than factors that predict new onset NSSI [6].

Further, NSSI research investigating social factors has often focused on a specific type of interpersonal context, such as peer victimization, without concomitantly studying other important relationship contexts, such as engagement with parents. This is potentially problematic, given research suggesting unique patterns of peer and parent effects on related types of psychopathology among youth. For example, research investigating quality of attachment to parents and peers simultaneously suggests that adolescent depression is directly related to poor attachment to parents, but only indirectly associated with poor attachment to peers [44].

To address these gaps in the literature, we investigated the effect of temporally prior parent and peer relationship characteristics on subsequent onset of NSSI among adolescent girls participating in an ongoing longitudinal study [45]. We chose to focus our investigation on four domains of interpersonal functioning that have been previously explored in relation to NSSI: (1) caregiver behaviors, such as punishment and praise [46, 47]; (2) caregiver-child relationship qualities, such as quality of attachment to parent [48]; (3) overt problems with peers, such as victimization [27]; and (4) intrapersonal risk factors for poor peer relationships, such as negative views of peers or one’s own social competence [49]. We specifically investigated how NSSI is associated with both behavioral and cognitive/affective indicators of relationship functioning for peer and family relationship domains. We tested the extent to which these interpersonal predictors, assessed yearly from 13 to 16, contributed to new onset NSSI during the following year, at ages 14–17.

Based on prior research in these areas, we hypothesized that harsh punishment, poor quality of attachment to the primary caregiver/parent, negative views of peers, and peer victimization would increase the odds of new onset NSSI. Although prior work has not investigated perceptions of one’s own social skills or social worth in relation to NSSI, we hypothesized that negative self-perceptions related to peer social functioning would increase the likelihood of new onset NSSI, given the strong association between self-directed negative emotions, self-criticism, and NSSI [50, 51]. Due to limited prior work investigating NSSI as it relates to nonviolent discipline, positive parenting behaviors, and parental monitoring, we did not develop a priori hypotheses for these constructs.

## Methods

### Participants and procedures

Data were drawn from the Pittsburgh Girls Study (PGS), an ongoing, longitudinal cohort study following a sample of girls (*N *= 2450) from childhood through adolescence. Detailed description of the recruitment and assessment procedures used in PGS is available elsewhere [45]. Briefly, four age cohorts of youth were enrolled in the study, along with their primary caregiver, at ages 5 through 8. Participants living in low-income city neighborhoods were oversampled, such that neighborhoods with at least 25% of families living at or below the federal poverty level were fully enumerated; a random selection of 50% of households were enumerated in all other neighborhoods. Participants have been assessed yearly since the study began in 2000. At each assessment, trained non-clinician staff administered a battery of self-report questionnaires as computer-assisted interviews. These standardized, in-home interviews were conducted with participants and their caregivers separately.

Lifetime and past-year NSSI were first assessed as part of the PGS battery when girls completed their age 13 assessment. Subsequent yearly assessments included evaluation of past-year NSSI. In order to evaluate antecedent predictors of NSSI onset, participants who reported a lifetime history of NSSI at their age 13 assessment were excluded from analyses, as information on age of NSSI onset was not available. A total of 2127 participants (97% of those interviewed at age 13) reported no lifetime history of NSSI at age 13 and were included in these analyses. Participants retained for analysis did not differ from those excluded on the basis of missing age 13 NSSI data or reported NSSI onset prior to age 13 with respect to age cohort, caregiver age at enrollment, caregiver gender, or caregiver relationship to child (coded as biological parent or other relationship; see Table 1 for descriptive characteristics). White participants were more likely to have missing data for age 13 NSSI (χ^2^(1) = 12.57, *p *< 0.001); there was, however, no relationship between race and history of NSSI reported at age 13 among those with age 13 NSSI data (χ^2^(1) = 2.18, *p *= 0.14).

**Table 1:** Descriptive characteristics of included sample (*N *= 2127)

	*n* (%) or *M* (*SD*)
Enrollment characteristics	
Cohort 5	506 (23.79)
Cohort 6	545 (25.62)
Cohort 7	542 (25.48)
Cohort 8	534 (25.11)
Caregiver characteristics	
Biological parent	1970 (92.62)
Other relationship	157 (7.38)
Female gender	1976 (92.90)
Household characteristics at age 13	
Household poverty	784 (36.86)
Single parent household	924 (43.44)
Participant race	
European–American/white	821 (42.30)
Minority race	1120 (57.70)

Caregivers were almost exclusively biological, adoptive, step, or foster parents (*n *= 2059, 97%), with the largest group being participants’ biological mothers (*n *= 1830, 86%). Therefore, we will use the term parent in the current manuscript. Girls were primarily of African–American (56%) or white/European–American (42%) descent; 60% of girls were identified as minority race (biracial, multiracial, and/or any race other than white). At the age 13 assessment, 43% (*n *= 924) of girls lived in a single parent household, and 37% (*n *= 784) of dyad households received some form of public assistance.

### Measures

#### Background and demographic information

Parents provided information on the girls’ race and household characteristics, such as whether both parents or a single parent lived in the home. They also reported on household poverty (yes/no) based on household receipt of any public assistance tied to low income (e.g. Temporary Aid for Needy Families, Medicaid, Women, Infants, and Children program).

#### Non-suicidal self-injury (NSSI)

Adolescent girls were first asked about NSSI at their age 13 assessment within the context of a structured interview administration of the Adolescent Symptom Inventory-4 [52], a measure of psychiatric symptoms. At that time, girls responded to the question, “Have you ever tried to hurt yourself even if you weren’t trying to kill yourself, like burning or cutting yourself?” At that assessment and at each subsequent yearly assessment, adolescents responded to the same question phrased as: “in the past year, have you…” to assess NSSI in the preceding year. Of those participants who reported no lifetime history of NSSI at age 13 (*n *= 2127), 44 (2.1%) subsequently reported new onset NSSI at age 14, 44 (2.1%) at age 15, 29 (1.5%) at age 16, and 20 (1%) at age 17.

It is plausible that, due to the ambiguous nature of the wording of this item, that some participants with a history of a suicide attempt, but without a history of NSSI, could respond affirmatively, leading to some lack of precision in our NSSI onset variable. To address this, we investigated the overlap of “yes” responses to this item with responses to another item that specifically assessed suicide attempts. Only 7 (5.3%) participants who were coded as having new onset NSSI also reported a suicide attempt by age 17, and of these, 6 reported multiple episodes of self-injurious behavior over a 1-year period, which is more consistent with NSSI than with attempted suicide. Further, research suggests that NSSI typically precedes suicide attempts temporally in adolescents and in nonclinical populations [53, 54].

#### Depression severity

Girls’ self-reported past year depressive symptom severity was assessed with the Adolescent Symptom Inventory-4 [52], a DSM-IV symptom checklist for emotional and behavioral disorders in youth. Symptoms were rated on four-point scales (0 = *never* to 3 = *very often*), with the exception of changes in appetite, sleep, activity, and concentration, which were scored as absent (0.5) or present (2.5). The sum of symptom scores was used as a measure of depression severity at each assessment. Girls with new onset NSSI at each assessment had significantly higher self-reported depressive symptom severity at the prior assessment than girls without new onset NSSI (all *p*_s_ < 0.05). The depression severity score showed good internal consistency reliability for assessments at ages 14 through 17 (Cronbach’s α = 0.79–0.84).

#### Parenting behaviors

Exposure to nonviolent discipline and harsh punishment was assessed using the Conflict Tactics Scale: Parent–Child version [55]. Adolescents rated ten items on a 3-point scale (1 = *never* to 3 = *often*) regarding the use of various types of discipline used by their parent. Four items assessing nonviolent discipline (explaining why the child’s behavior was wrong, using time-out, distracting the child, or stopping privileges) exhibited adequate internal consistency across ages 13–16 in this sample (Cronbach’s α = 0.64–0.66). Harsh punishment was assessed by combining five items measuring psychological aggression (shouting, swearing, or name-calling directed at the child, threatening to kick the child out of the home, or threatening to hit the child) with a single item assessment of spanking. This construct exhibited adequate internal consistency (Cronbach’s α = 0.75–0.77).

The Positive Parenting Scale [56] includes seven items assessing encouraging behaviors directed towards the child rated on a three-point scale (1 = *almost never* to 3 = *a lot).* Youth rated how often their parent did a variety of affirming behaviors when they did something the parent liked, such as providing verbal praise or giving hugs. Internal consistency reliability was good (Cronbach’s α = 0.83–0.86).

Four items from the Supervision Involvement Scale [56] were used to assess parental monitoring (e.g., “Do your parent(s) know who you are with when you are away from home?”). Youth rated these items on a three-point scale (1 = *almost always* to 3 = *almost never*). Reliability for this scale was adequate (Cronbach’s α = 0.63–0.68) across ages 13–16.

#### Quality of attachment to parent

Girls completed the trust subscale of the Revised Inventory of Parent and Peer Attachment [57], a simplified version of the Inventory of Parent and Peer Attachment [37]. The trust subscale is comprised of ten items assessing adolescents’ perception of their parent’s availability, sensitivity, understanding, and sense of mutual respect, and provides an indicator of quality of attachment to one’s parent. One item (“My parents expect too much from me”) was removed from the scale, as it had the lowest factor loading and lowest item-total correlation in earlier studies [58]. The remaining nine items were scored on a three-point scale (1 = *never true* to 3 = *always true*); some items were reverse coded. Items were coded such that higher values indicated poorer attachment. The internal consistency of the sum of item scores was high across ages 13–16 (Cronbach’s α = 0.89–0.92).

#### Peer and social self-perceptions

Girls completed the revised Perceptions of Peers and Self Inventory [59, 60], which measures youths’ social-cognitive perceptions of their peers, as well as of themselves in relation to others. The perceptions of peers subscale includes 15 items assessing children’s perceptions of their peers and friendships (e.g., “Other kids will try to put you down or tease you if they have a chance”). The social self-worth subscale includes eight items assessing adolescents’ feelings about their ability to be a good friend (e.g., “It’s a waste of other kids’ time to be friends with me”). The social self-competence subscale is comprised of seven items assessing children’s appraisals of their own social skills (e.g., “I am not very good at getting other kids to let me join in their games”). These self-reports are associated with observer ratings of child social behavior and child popularity [59, 60]. All items were scored on a four-point scale (1 = *not at all* to 4 = *very much*); some items were reverse scored, such that, for all items, higher scores indicated more negative views of peers and of adolescents’ own social value and competence. Internal consistency for the subscales at ages 13 through 16 was highest for perceptions of peers (Cronbach’s α = 0.78–0.80), then social self-worth (Cronbach’s α = 0.72–0.73), and poorest for social self-competence (Cronbach’s α = 0.52–0.54).

#### Peer victimization

Girls provided data on their experiences of peer victimization on the Peer Victimization Scale [61]. Nine items assessed frequency of victimization by verbal aggression, physical aggression, and ostracism over the preceding 3 months, rated on five-point scales (0 = *never* to 4 = *a few times a week*). Item scores were summed to create a composite measure of recent peer victimization. This measure shows good reliability at ages 13 to 16 in this sample (Cronbach’s α = 0.76–0.79).

### Data analytic strategy

We conducted a series of discrete-time (person-year) survival analyses [62] to model time-variant and time-invariant predictors of NSSI onset at ages 14, 15, 16, and 17. Discrete-time survival analyses account for dependency across repeated measures within individuals, as well as for the modeling of time-lagged predictors of the outcome of interest at each assessment. Analyses were conducted in Mplus version 8.1 [63] using a logit-link function and maximum likelihood estimation with robust standard errors. To account for missing data on the observed predictor and covariate measures, these variables were brought into the model using Monte Carlo numerical integration.

**Fig. 1:**
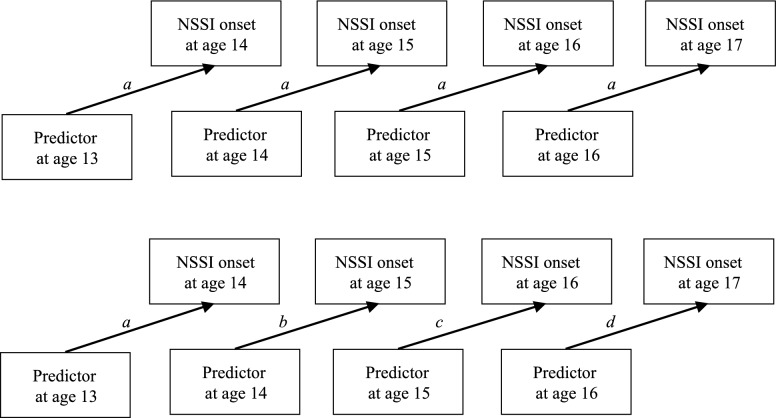
Path diagram of proportional and nonproportional discrete-time survival models. Top figure shows a proportional model, in which the time-lagged associations between predictors at age *t* and NSSI onset at age *t *+ *1* are set to equality across all assessment waves. Bottom figure shows a non-proportional model, in which each time-lagged association is estimated independently, and can vary over time

Discrete-time survival analyses can be modeled holding the effects of time-varying predictors constant across time (proportional models) or allowing these effects to vary over time (nonproportional models; see Fig. 1 for diagrammatic representation). For example, in a proportional model, the time-lagged effect of age 13 depression symptoms on age 14 NSSI would be held equal to the effect of age 14 depressive symptoms on age 15 NSSI, as well as to the effect of age 15 depressive symptoms on age 16 NSSI, and to age 16 depressive symptoms on age 17 NSSI. In a nonproportional model, these effects would be permitted to vary based on observed relationships between the data at each age. In both types of models, the effects of time-invariant predictors, such as racial background, are modeled as having a proportional (equivalent) effect across time. For each analysis described below, parallel proportional and nonproportional models were compared using a χ^2^ difference test (Δχ^2^) based on loglikelihood values and scaling correction factors. For analyses in which the nonproportional (less constrained) model did not exhibit significantly improved fit than the proportional (more constrained, i.e., more parsimonious) model, we present results from the proportional analysis.

Prior to conducting our analyses of interest, we tested the effects of relevant time-invariant characteristics as potential covariates. Specifically, we tested a model in which minority race, cohort, and household poverty and single parent status at age 13 predicted NSSI onset at ages 14 through 17. All covariates were coded as binary except for cohort, which was ordinal (for the cohorts beginning participation in the PGS at ages 5, 6, 7, and 8). Based on the relationship between depressive symptom severity and NSSI in our data, as well as the established relationship between depression and NSSI in adolescents more generally [7, 46, 47], we included depressive symptom severity from the prior year as a predictor of next-year NSSI onset in our analyses.

After determining covariates for inclusion in our analyses, we tested a series of models to evaluate the relationships between parent and peer relationship characteristics and NSSI onset. First, we evaluated each independent variable as a predictor of NSSI in separate models, each including covariates. Second, we tested a parent factors model, including all parent relationship indicators that were significantly associated with NSSI in the first set of models, and a peer factors model, including all significant peer relationship predictors of NSSI from earlier models. Third, we tested a combined model in which significant parent and peer relationship indicators were investigated simultaneously as predictors of NSSI onset. Although some of these constructs are moderately correlated with each other (see Table 2), tests of multicollinearity yielded variance inflation factor values between 1 and 2.1, suggesting that multicollinearity is unlikely to cause significant problems in our models predicting new onset NSSI.

**Table 2:** Correlation matrix of NSSI predictors at age 13

	*M* (*SD*)	HP	ND	QA	PP	PM	SSC	SSW	POP	PV
HP	8.74 (2.30)	1								
ND	7.27 (1.88)	− 0.07	1							
QA	11.45 (3.20)	0.42	0.23	1						
PP	16.59 (3.21)	− 0.24	− 0.36	− 0.54	1					
PM	4.71 (1.17)	0.18	0.21	0.29	− 0.24	1				
SSC	11.45 (2.59)	0.10	0.07	0.15	− 0.25	0.15	1			
SSW	11.76 (2.91)	0.11	0.13	0.23	− 0.26	0.14	0.56	1		
POP	25.99 (5.68)	0.26	0.05	0.23	− 0.21	0.19	0.50	0.58	1	
PV	2.70 (3.69)	0.23	− 0.03	0.18	− 0.09	0.12	0.29	0.32	0.45	1
DEP	6.97 (4.47)	0.30	0.06	0.33	− 0.16	0.18	0.20	0.22	0.30	0.40

*p *< 0.05 for values ≥ |0.05|, *p* < 0.01 for values ≥ |0.06|, *p *< 0.001 for values ≥ |0.09|. Correlation matrix for predictors assessed at ages 14, 15, and 16 available upon request from the corresponding author

*HP* harsh punishment, *ND* nonviolent discipline, *QA* (poor) quality of attachment to parent, *PP* positive parenting, *PM* (low) parental monitoring, *SSC* social self-competence, *SSW* social self-worth, POP (negative) perceptions of peers, *PV* peer victimization, *DEP* depression severity

## Results

### Time-invariant and time-varying covariates

Across ages 14–17, NSSI onset was significantly associated with race (*OR *= 0.59, 95% CI [0.39, 0.90], *p *= 0.01), indicating that girls of minority racial background were less likely to experience NSSI onset during this timeframe compared to white girls. There was also evidence of a cohort effect, such that girls enrolled at older ages in assessment wave 1 were more likely to report subsequent NSSI (*OR *= 1.18, 95% CI [1.01, 1.38], *p *= 0.04). There were no significant relationships between household poverty or single parent status and NSSI onset. For depression severity as a time-varying predictor of NSSI onset, the χ^2^ difference test indicated no significant differences in model fit between proportional and nonproportional models (Δχ^2^ [3] = 3.88, *p* = 0.28), indicating that the effect of depression severity on odds of next-year NSSI onset (which was significant in each year, *p*s < 0.003) did not vary over time. Hence, these paths were constrained to equality in subsequent models. All subsequent models included minority race and cohort as time-invariant predictors of NSSI onset, in addition to time-varying depression severity.

### Univariate models of parent and peer factors and NSSI

In a series of models that included minority race, cohort, and depression severity, we investigated the contribution of each parent and peer relationship factor to new onset NSSI separately. In all but one case (for nonviolent discipline), the χ^2^ difference test indicated no significant improvement in model fit for nonproportional models, suggesting that effects of most parent and peer relationship factors did not vary with age. Therefore, proportional model results, holding the effects of each predictor constant over time, are presented below for all predictors except nonviolent discipline.

Harsh punishment was positively associated with subsequent NSSI onset (*OR *= 1.10, 95% CI [1.02, 1.17], *p *= 0.008), as was poor quality of attachment to the parent (*OR *= 1.07, 95% CI [1.02, 1.11], *p *= 0.002). Low parental monitoring was associated with increased odds of NSSI onset during the following year (*OR *= 1.15, 95% CI [1.02, 1.31], *p *= 0.03), whereas positive parenting predicted decreased likelihood of subsequent NSSI onset (*OR *= 0.94, 95% CI [0.89, 0.99], *p *= 0.01. In a nonproportional model, nonviolent discipline was not associated with subsequent NSSI onset at any age.

All indicators of peer interpersonal difficulties were predictive of next year NSSI onset. This effect was similar in magnitude for peer victimization (*OR *= 1.08, 95% CI [1.05, 1.12], *p *< 0.001), negative perceptions of peers (*OR *= 1.05, 95% CI [1.01, 1.08], *p *= 0.007), social self-worth (*OR *= 1.11, 95% CI [1.05, 1.17], *p *< 0.001), and social self-competence (*OR *= 1.08, 95% CI [1.01, 1.15], *p *= 0.03).

### Parental behaviors and parent relationship characteristics

Based on results from earlier analyses, we subsequently evaluated a combined model in which harsh punishment, quality of attachment to parent, and poor parental monitoring were evaluated as predictors of following-year NSSI onset, controlling for covariates (see Table 3). In this combined model, the χ^2^ difference test again indicated no significant improvement with the nonproportional model, in which effects were allowed to vary over time, compared to the proportional model, in which effects were fixed to equality (Δχ^2^ [12] = 12.13, *p* = 0.44), favoring the more parsimonious proportional model. Results of the combined proportional model demonstrated that none of the parent relationship indicators that were significant in the univariate analyses retained a significant association with following year NSSI onset when they were evaluated jointly. This suggests that, while parent relationship factors may contribute to NSSI onset generally, none of the constructs included here exhibited *unique* relationships with subsequent NSSI, controlling for the effects of other parent relationship factors.

**Table 3:** Discrete-time survival model of NSSI onset and parent relationship factors

	Estimate (*b*)	Standard error (*SE*)	*p* value	Logistic *OR* [95% CI]
Minority status	− 0.78	0.18	< 0.001	0.46 [0.32, 0.66]
Cohort	0.11	0.08	0.18	1.11 [0.95, 1.30]
Depression severity	0.11	0.02	< 0.001	1.12 [1.08, 1.16]
Harsh punishment	0.06	0.04	0.11	1.06 [0.99, 1.14]
(Poor) quality of attachment to parent	0.03	0.03	0.39	1.03 [0.97, 1.09]
Positive parenting	− 0.03	0.03	0.40	0.97 [0.91, 1.04]
(Low) parental monitoring	0.10	0.06	0.11	1.11 [0.98, 1.25]

### Perceptions of peers and peer relationship characteristics

We next tested a model in which girls’ experiences with and views about peers, as well as their perceptions of themselves in relationship to peers, predicted subsequent NSSI onset (see Table 4). Results of the χ^2^ difference test again favored the more parsimonious proportional model (Δχ^2^ [12] = 12.87, *p* = 0.38). In this combined model, negative perceptions of peers were not significantly associated with next-year NSSI onset (*OR *= 1.00, *p* = 0.93), whereas peer victimization was positively associated with NSSI onset during the following year (*OR *= 1.07, *p *= 0.001). Poor social self-worth was also significantly associated with odds of subsequent new onset NSSI (*OR *= 1.09, *p *= 0.01). In contrast, perceived competence in social situations was not associated with later NSSI onset (*OR *= 0.99, *p *= 0.87).

**Table 4:** Discrete-time survival model of NSSI onset and peer relationship factors

	Estimate (*b*)	Standard error (*SE*)	*p* value	Logistic *OR* [95% CI]
Minority status	− 0.63	0.19	0.001	0.53 [0.37, 0.78]
Cohort	0.13	0.08	0.13	1.13 [0.97, 1.33]
Depression severity	0.10	0.02	< 0.001	1.11 [1.07, 1.15]
(Negative) perceptions of peers	0.00	0.02	0.93	1.00 [0.96, 1.04]
(Low) social self-worth	0.09	0.04	0.01	1.09 [1.02, 1.17]
(Low) social self-competence	− 0.01	0.05	0.87	0.99 [0.90, 1.09]
Peer victimization	0.07	0.02	0.001	1.07 [1.03, 1.11]

### Omnibus model of parent and peer predictors of NSSI

**Fig. 2:**
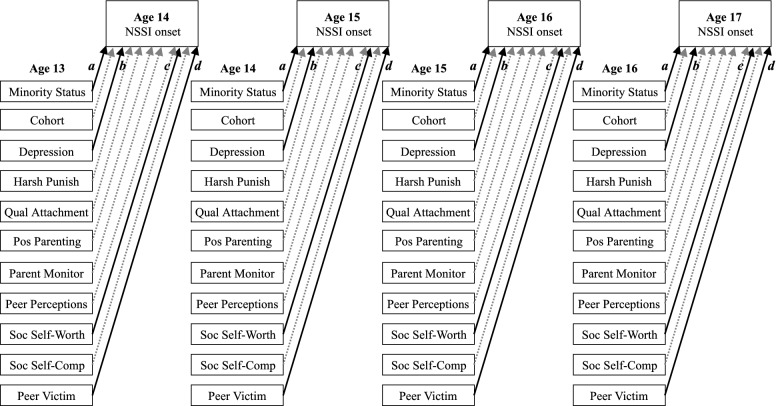
Path diagram of proportional discrete-time survival model of NSSI onset and both parent and peer relationship factors. Coefficients *a, b, c,* and *d* represent the significant proportional effects from the final omnibus model (see Table 5 for additional information). Paths displayed using dotted grey arrows were not statistically significant

For the omnibus parent and peer predictors model, we included all indicators that exhibited a significant association with NSSI onset in earlier univariate models (e.g., all tested variables with the exception of nonviolent discipline; see Fig. 2 and Table 5). Results of the χ^2^ difference test favored the more parsimonious, proportional model (Δχ^2^ [24] = 26.71, *p *= 0.32), which is presented here. As in the parent factors only model, no parent relationship characteristic had a significant, unique association with following year NSSI onset in the omnibus model. Similar to the peer factors only model, neither social self-competence nor perceptions of peers were associated with subsequent new onset NSSI. Both social self-worth and peer victimization, however, retained significant associations with later NSSI onset, such that poorer social self-worth (*OR *= 1.08, *p *= 0.02) and higher frequency of peer victimization (*OR *= 1.07, *p *= 0.001) at ages 13–16 predicted increased odds of new onset NSSI in the following year.

**Table 5:** Discrete-time survival model of NSSI onset and both parent and peer relationship factors

	Estimate (*b*)	Standard error (*SE*)	*p* value	Logistic *OR* [95% CI]
Minority status	− 0.70	0.19	< 0.001	0.50 [0.34, 0.73]
Cohort	0.12	0.08	0.15	1.13 [0.96, 1.32]
Depression severity	0.09	0.02	< 0.001	1.09 [1.05, 1.14]
Harsh punishment	0.05	0.04	0.16	1.05 [0.98, 1.14]
(Poor) quality of attachment to parent	0.02	0.03	0.60	1.02 [0.96, 1.08]
Positive parenting	− 0.03	0.03	0.37	0.97 [0.91, 1.04]
(Low) parental monitoring	0.08	0.06	0.23	1.08 [0.95, 1.22]
(Negative) perceptions of peers	− 0.01	0.02	0.62	0.99 [0.95, 1.03]
(Low) social self-worth	0.08	0.04	0.02	1.08 [1.01, 1.16]
(Low) social self-competence	− 0.01	0.05	0.91	0.99 [0.90, 1.09]
Peer victimization	0.07	0.02	0.001	1.07 [1.03, 1.11]

## Discussion

The current study evaluates the time-lagged associations between both peer and parent relationship characteristics and new onset NSSI in a large, urban community sample of adolescent girls. This approach addresses many of the limitations of extant research, including the use of cross-sectional designs, a focus on specific interpersonal domains in isolation from each other, and the conflation of correlates of NSSI with predictors of NSSI onset.

Among girls without history of NSSI at age 13, NSSI onset at ages 14 through 17 was more likely for girls who reported high levels of harsh punishment by their parent. This is consistent with prior research suggesting that harsh punishment may be associated with continued NSSI or a history of NSSI, particularly for girls [36, 38], and extends these findings by showing that harsh punishment is also a risk factor for new onset of NSSI in adolescence. Poor quality of attachment to the parent also predicted following-year NSSI onset, which extends prior cross-sectional research in this domain [30]. In contrast to earlier cross-sectional research focused on history of any NSSI [31], we also found that low parental monitoring of youths’ behaviors predicted increased odds of subsequent NSSI onset. This suggests that poor monitoring heightens risk for NSSI initiation, but is unrelated to continued engagement in NSSI. Importantly, our results highlight the protective effects of positive parenting behaviors in reducing the odds of NSSI onset over the following year. In each of these analyses, significant effects were found for parent behavior and cognitive/affective relationship characteristics, *above and beyond* the effect of depression severity and other covariates (such as minority race).

Although these parent relationship characteristics were each significantly associated with subsequent new onset NSSI in individual models, no single parent relationship construct exhibited a significant unique association with later NSSI when other parent-related variables were included in a combined multivariate model. This may be due in part to shared method variance, as all predictors were based on adolescents’ report. This may also suggest that parent–child relationship factors in general, rather than any specific facet of parenting or parent–child relationships, can contribute to or protect against NSSI.

With respect to peer functioning, we tested how adolescents’ general views about peers, specific experiences with peers, and views of themselves in relation to other adolescents related to new onset NSSI, above and beyond the effects of depression severity, race, and cohort. As expected, both frequency of peer victimization over a 3-month period and negative beliefs about peers were positively associated with new onset NSSI. In the combined model, however, only peer victimization predicted later NSSI onset; this is noteworthy, given that negative views of peers is associated with less popularity and more peer problems among youth [59]. This pattern may indicate that more readily observable, behavioral indicators of peer problems are more strongly predictive of NSSI than one’s interpretations or beliefs about these experiences. Additionally, although both poor social self-worth and poor social self-competence predicted increased odds of NSSI onset independently, only social self-worth, continued to exhibit a unique association with later NSSI onset in the combined peer relationship characteristics model.

These patterns of results may be explained in several ways. It is possible that peer victimization and poor social self-worth are especially pernicious with respect to adolescent psychopathology and emotional health, and that these experiences therefore have unique associations with later NSSI. It is also possible that peer victimization negatively influences social self-worth, or that impaired self-worth increases risk for peer victimization, such that these factors reinforce each other, magnifying the independent effects on subsequent NSSI. Further, prior research demonstrates an association between self-criticism and both peer victimization [64] and poor social self-worth [65]; these effects, therefore, may indicate an underlying risk for self-criticism, which is robustly associated with NSSI [50, 66–68].

In addition to our parent and peer relationships findings, and consistent with prior research [69, 70], we found that girls of minority racial or ethnic background (primarily African-American), had lower odds of NSSI onset during adolescence than girls of European American descent. Although further research is needed to examine the potential mechanisms contributing to these group differences, there is some evidence to suggest that reduced risk of NSSI among African-American youth may be related to a sense of ethnic identity or belonging [70].

As with any type of research, this study has several strengths, as well as limitations. First, our assessment of NSSI was based on a single item which asked participants about hurting themselves “even if” they were not attempting to kill themselves. Although we believe that the likelihood of miscategorizing participants on the basis of attempted suicide, but not NSSI, is relatively low (see Methods, above), we cannot rule out this possibility entirely. Further, we were unable to reliably investigate other aspects of NSSI phenomenology, such as specific NSSI methods and overall NSSI frequency, which precludes us from determining the severity or chronicity of NSSI among youth who endorsed NSSI onset.

Because these data are drawn from a large, longitudinal community cohort study (PGS), we were able to follow a large enough sample of individuals to appropriately model new onset NSSI, as well as to evaluate the temporal precedence of our predictors and outcomes of interest. It is, however, likely that other, unmeasured variables also occur prior to NSSI onset, and may play a role in the development of NSSI. Consistent with the role of other processes in NSSI onset, the magnitude of our significant effects was quite small (largest OR = 1.11), highlighting the need to investigate other types of risk factors for NSSI onset. In order to address one such additional factor, all our analyses included time-lagged depression severity as a covariate, such that all our results are based on associations with new onset NSSI above and beyond the effect of depressive symptoms on later NSSI. Further, we chose to limit our analyses to participants who reported no lifetime history of NSSI at age 13, the first year in which participants were asked about NSSI, to ensure that subsequent endorsement of NSSI was truly an indicator of NSSI onset; this improved our ability to make inferences specifically about new engagement in NSSI, but also limits interpretation to only adolescents who first begin NSSI at age 14 or later, who may differ from adolescents who begin NSSI at earlier ages. Further, although the ability to identify antecedent indicators of risk for NSSI onset is novel, our study cannot speak to the factors that contribute to NSSI recovery [71], for instance, the role of family functioning in recovery among youth [72].

Our results are limited to associations among females. As NSSI appears to be somewhat more common among women [73], understanding these associations has high clinical utility; however, future research will need to investigate the extent to which these findings generalize to adolescent boys, as well as to individuals who do not identify as cisgender. Additionally, this sample was predominantly African-American and white, and entirely recruited from the Pittsburgh metropolitan area. Although we controlled for racial minority status in our analyses, the minority race group was predominantly comprised of African-Americans (see Table 1), limiting our ability to make inferences about individuals who identify with other minority racial groups, for example, Asian-American. It will be important to determine whether and how our results change when investigated in other racial or ethnic groups.

In spite of these limitations, our findings provide valuable insight into the roles of parent and peer relationships in development of NSSI during adolescence. They highlight the importance of assessing interpersonal functioning, and the need to consider multiple aspects of family and peer relationships, rather than investigating a single component of these complex dynamics as a predictor of NSSI. Our results suggest that, for adolescent girls, experiences of peer victimization and poor social self-worth may elevate risk for subsequent development of NSSI over and above other important risk factors such as depression severity and family context.

Although some early intervention programs exist targeting youth NSSI [74], they are focused on motivating help-seeking among those already engaging in NSSI, rather than on preventing NSSI before it begins. By improving our understanding of early indicators of risk for NSSI onset, our results have implications for the development of NSSI prevention programs targeted at high-risk adolescent girls. These programs could focus, for example, on responding effectively to bullying and relational victimization, or on developing positive views of the self. Notably, there is preliminary evidence that self-criticism, which is associated with poor self-worth, can be attenuated through relatively brief interventions [66, 75]. Although these interventions do not yet have evidence for their effectiveness in actually reducing NSSI behaviors among those who already engage in NSSI [75], these programs may hold benefit for at-risk youth who have not yet begun to engage in NSSI.

## Abbreviations

CI: confidence interval
NSSI: non-suicidal self-injury
OR: odds ratio
PGS: Pittsburgh Girls Study
